# Phosphodiesterase 4B negatively regulates endotoxin-activated interleukin-1 receptor antagonist responses in macrophages

**DOI:** 10.1038/srep46165

**Published:** 2017-04-06

**Authors:** Jing-Xing Yang, Kou-Chou Hsieh, Yi-Ling Chen, Chien-Kuo Lee, Marco Conti, Tsung-Hsien Chuang, Chin-Pyng Wu, S.-L. Catherine Jin

**Affiliations:** 1 Department of Life Sciences, National Central University, Zhongli District, Taoyuan City, Taiwan, Republic of China; 2 Internal Medicine, Landseed Hospital, Pingzhen District, Taoyuan City, Taiwan, Republic of China; 3 Graduate Institute of Immunology, National Taiwan University College of Medicine, Taipei, Taiwan, Republic of China; 4 Department of Obstetrics, Gynecology, and Reproductive Sciences, University of California San Francisco, California, USA; 5 Immunology Research Center, National Health Research Institutes, Miaoli, Taiwan, Republic of China

## Abstract

Activation of TLR4 by lipopolysaccharide (LPS) induces both pro-inflammatory and anti-inflammatory cytokine production in macrophages. Type 4 phosphodiesterases (PDE4) are key cAMP-hydrolyzing enzymes, and PDE4 inhibitors are considered as immunosuppressors to various inflammatory responses. We demonstrate here that PDE4 inhibitors enhance the anti-inflammatory cytokine interleukin-1 receptor antagonist (IL-1Ra) secretion in LPS-activated mouse peritoneal macrophages, and this response was regulated at the transcriptional level rather than an increased IL-1Ra mRNA stability. Studies with PDE4-deficient macrophages revealed that the IL-1Ra upregulation elicited by LPS alone is PKA-independent, whereas the rolipram-enhanced response was mediated by inhibition of only PDE4B, one of the three PDE4 isoforms expressed in macrophages, and it requires PKA but not Epac activity. However, both pathways activate CREB to induce IL-1Ra expression. PDE4B ablation also promoted STAT3 phosphorylation (Tyr^705^) to LPS stimulation, but this STAT3 activation is not entirely responsible for the IL-1Ra upregulation in PDE4B-deficient macrophages. In a model of LPS-induced sepsis, only PDE4B-deficient mice displayed an increased circulating IL-1Ra, suggesting a protective role of PDE4B inactivation *in vivo*. These findings demonstrate that PDE4B negatively modulates anti-inflammatory cytokine expression in innate immune cells, and selectively targeting PDE4B should retain the therapeutic benefits of nonselective PDE4 inhibitors.

The production of pro-inflammatory cytokines by the immune system is crucial in the host’s defense against infection. The activities of these cytokines, however, must be tightly regulated *in vivo* to prevent detrimental effects. For instance, overproduction of IL-1 in local tissues is implicated in autoimmune diseases such as rheumatoid arthritis^1^, and systemic elevation of pro-inflammatory cytokines, including TNF-α and IL-1, is involved in endotoxin-induced septic shock^2^. It is well recognized that in addition to pro-inflammatory actions, the immune system also promotes various negative feedback mechanisms and anti-inflammatory signals to avoid excessive inflammation^3^. Among the anti-inflammatory mediators studied, interleukin-1 receptor antagonist (IL-1Ra) has drawn particular attention for its clinical significance in certain inflammatory diseases. IL-1Ra is a member of the IL-1 family cytokines that binds to IL-1 receptors (IL-1R) on target cells to abrogate the inflammatory effects of IL-1, but not elicit downstream signaling^4^. The human recombinant IL-1Ra anakinra currently is used as a therapeutic agent for the treatment of rheumatoid arthritis.

Both IL-1 and IL-1Ra are produced concomitantly in immune cells in response to various inflammatory stimuli, such as microbial products and pro-inflammatory cytokines^5^,^6^. IL-1Ra is expressed as secretory (sIL-1Ra) and intracellular (icIL-1Ra) forms, and both bind with high affinity to IL-1R1 to antagonize the effects of IL-1. The intracellular isoforms serve as a reservoir of IL-1Ra that are released upon cell death or actively secreted by an unknown pathway, aiding to confine the inflammation of tissue damage^7^. The secretory IL-1Ra is produced primarily in immune cells and is able to downregulate the production of several pro-inflammatory cytokines, such as endotoxin-induced TNF-α and IL-1β production^8^. Elevation of IL-1Ra is also observed in the serum or inflamed tissues of patients with certain inflammatory diseases^7^. It is generally accepted that the induction of IL-1Ra during inflammation is essential for preventing exaggerated immune responses as IL-1Ra deficiency has been shown to cause spontaneous development of arthritis and skin lesions reminiscent of psoriasis in mouse models^9^,^10^ as well as auto-inflammatory disorders, such as DIRA (deficiency of interleukin-1-receptor antagonist) in patients^11^,^12^. Given the importance of IL-1Ra in inflammatory diseases, it becomes pertinent to gain insights into the mechanisms underlying the regulation of its production. Such information may lead to the development of novel agents for the treatment of related inflammatory diseases.

The expression of IL-1Ra is strongly induced in monocytes and macrophages in response to lipopolysaccharide (LPS), an outer membrane component of Gram-negative bacteria. Through activation of toll-like receptor 4 (TLR4), LPS activates both MyD88- and TRIF-dependent signal pathways, leading to activation of several downstream cascades, including the NF-κB (nuclear factor κB), ERK1/2 (extracellular-signal regulated kinase 1/2), p38 MAPK (mitogen-activated protein kinase), and JNK (c-Jun N-terminal kinase) pathways, as well as the transcriptional factor IRF3 (interferon regulatory factor 3), which all contribute to the expression of inflammatory cytokines^13^. In addition to these major TLR4 signaling pathways and components, several other regulators capable of potentiating LPS-stimulated IL-1Ra production have also been identified. These include the cytokine IL-10^14^,^15^, phosphatidylinositide 3-kinases (PI3K)^16^, mitogen- and stress-activated kinase 1 (MSK1)^17^, glycogen-synthase kinase 3 (GSK3) inhibitors^18^, and cAMP elevators^19^. The second messenger cAMP is generally considered a negative modulator of a variety of inflammatory cell responses, including pro-inflammatory mediator generation and receptor-mediated phagocytosis, by activating its effectors protein kinase A (PKA) or exchange proteins directly activated by cAMP (Epac)^20^. However, we and others have demonstrated that the cAMP-elevating agents also promote IL-1Ra production in LPS-stimulated macrophages^19^,^21^, but the molecular mechanisms underlying this cAMP effect remained to be elucidated.

Phosphodiesterase 4 (PDE4) is a family of cAMP-hydrolyzing enzymes expressed in almost all immune and inflammatory cells, inferring their importance in regulation of intracellular cAMP level and hence, immune responses in these cells^22^,^23^. By elevating cAMP level, PDE4 inhibitors have been shown to suppress a myriad of inflammatory responses in most immune and inflammatory cells^22^,^23^. Moreover, PDE4 inhibitors are used as anti-inflammatory drugs for the treatment of inflammatory disorders, such as roflumilast for chronic obstructive pulmonary disease (COPD) and apremilast for psoriasis and psoriatic arthritis. While exerting beneficial effects, these inhibitors are associated with adverse effects such as nausea, emesis, and diarrhea, thereby limiting their dosing and clinical efficacy^24^,^25^. The PDE4 family consists of four genes (*PDE4A-D*). Among them, three (PDE4A, 4B, and 4D) are expressed in macrophages, in which LPS stimulation causes a major upregulation of the PDE4B isoform^26^,^27^. It has been postulated that the side effects with currently used PDE4 inhibitors probably is caused by their non-specific action across the different isoforms. Using the PDE4 gene targeting approach, we have demonstrated that certain inflammatory cell responses *in vitro* and *in vivo* are suppressed by ablation of PDE4B but not PDE4A or PDE4D^21^,^28^. To ensure that PDE4B is a useful target for the development of anti-inflammatory drugs to improve the therapeutic index of the nonselective PDE4 inhibitors, uncovering its essential role in additional inflammatory processes appears to be indispensable.

We previously demonstrated that treatment of Raw 267.4 macrophages with the PDE4 inhibitor rolipram enhances LPS-stimulated IL-1Ra secretion^21^. In the present study, we further explored the molecular mechanisms for this PDE4 regulation in mouse peritoneal macrophages. We found that the rolipram-enhanced IL-1Ra production in response to TLR4 activation is mediated by inhibition of PDE4B but not PDE4A or PDE4D, and ablation of PDE4B induces activation of the downstream cAMP-PKA rather than cAMP-Epac signal pathway. Moreover, both PKA-dependent and PKA-independent CREB activation were involved in the LPS-stimulated IL-1Ra production in PDE4B null macrophages. Although PDE4B ablation was found to increase STAT3-Tyr^705^ phosphorylation to LPS stimulation, this STAT3 activation only partly contributes to the IL-1Ra enhancement in rolipram-treated or PDE4B null macrophages.

## Results

### Inhibition of PDE4 upregulates LPS-induced IL-1Ra production in mouse macrophages

**Figure 1:**
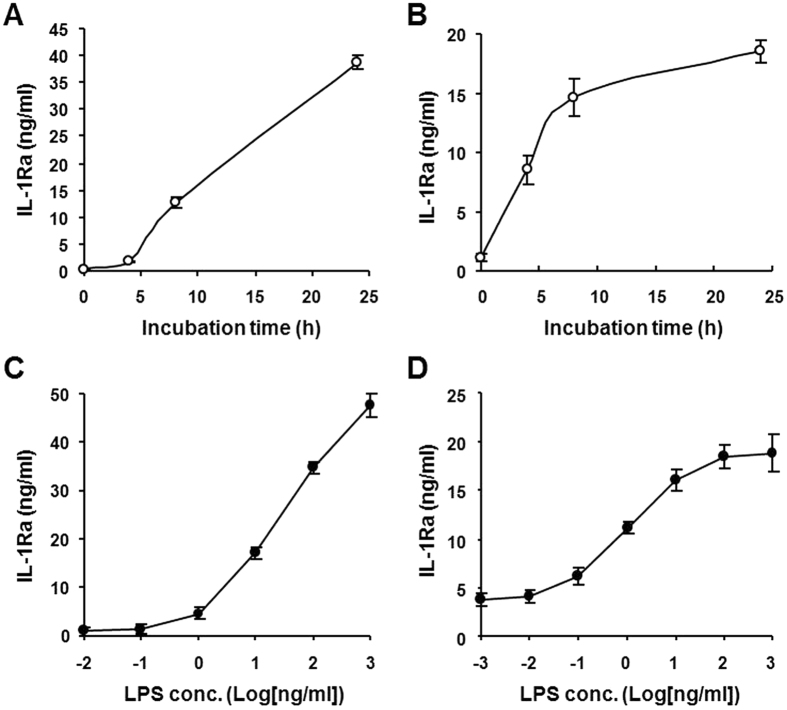
Time- and dose-dependent IL-1Ra secretion in LPS-stimulated mouse macrophages. Raw 264.7 cells (**A** and **C**) and mouse peritoneal macrophages (**B** and **D**) were incubated with LPS (10 ng/ml) for the indicated times (**A** and **B**) or increasing concentrations of LPS for 8 h (**C** and **D**). Accumulation of IL-1Ra in the medium was measured by ELISA. Data are the mean ± SEM (*n* = 5–6 in A; *n* = 7 in B and C; *n* = 9–13 in D).

Using a proteomic approach we previously demonstrated that the PDE4 inhibitor rolipram promotes IL-1Ra secretion in LPS-stimulated Raw 264.7 macrophages^21^. In this study, we further investigated the molecular mechanisms underlying the PDE4 regulation of IL-1Ra secretion in murine macrophages. To determine the optimal LPS stimulation conditions, initially Raw 264.7 and mouse peritoneal macrophages were incubated with LPS at different concentrations and times, and the levels of IL-1Ra in the culture medium were measured by ELISA. The results showed that in the presence of 10 ng/ml LPS, the IL-1Ra accumulation in the medium displayed a time-dependent increase up to 24 h for Raw 264.7 cells (Fig. 1A), whereas the increase in the primary macrophages reached nearly maximum at 8 h and plateaued thereafter (Fig. 1B). Additionally, both cells exhibited dose-dependent increase in IL-1Ra secretion to LPS stimulation, where the IL-1Ra response in Raw 264.7 cells continued to increase with the LPS concentration up to 1 μg/ml (Fig. 1C), while the maximal secretion of IL-1Ra was obtained at 10–100 ng/ml of LPS in the peritoneal macrophages (Fig. 1D).

**Figure 2:**
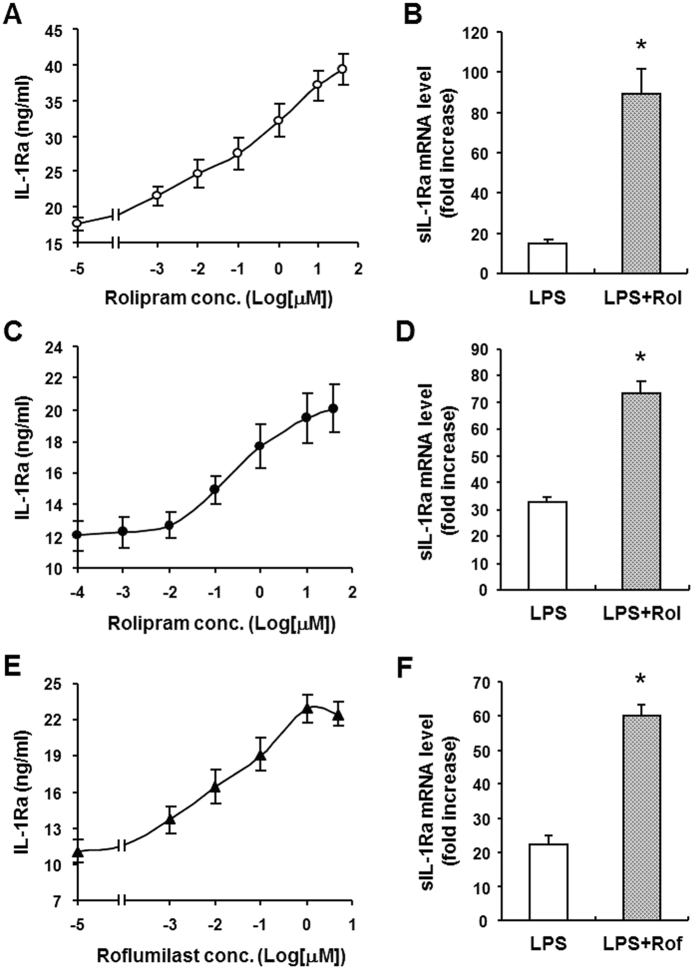
Effect of PDE4 inhibitor on LPS-induced IL-1Ra secretion and mRNA expression in mouse macrophages. Raw 264.7 cells (**A** and **B**) and mouse peritoneal macrophages (**C**–**F**) were pretreated for 20 min with increasing concentrations of rolipram (**A** and **C**) or roflumilast (**E**) or with 10 μM rolipram (**B** and **D**) or 1 μM roflumilast (**F**) before LPS (10 ng/ml) stimulation for 8 h (**A**,**C** and **E**) or 3 h (**B**,**D** and **F**). IL-1Ra accumulation in the medium was measured by ELISA (**A**,**C** and **E**). The secretory form IL-1Ra (sIL-1Ra) mRNA levels in the cells were determined by real-time PCR and expressed as fold increase to the untreated cells (**B**,**D** and **F**). Data are the mean ± SEM (*n* = 4–6 in A and B; *n* = 6–9 in C and D; *n* = 4–8 in E and F). *P < 0.001, compared with LPS control.

To confirm the rolipram effect on IL-1Ra production, Raw 264.7 macrophages were treated with 10 ng/ml LPS for 8 h in the presence of increasing concentrations of rolipram. Figure 2A shows that the IL-1Ra secretion was dose-dependently increased with rolipram, an observation confirming our previous proteomics data^21^. Further real-time PCR analysis revealed that these cells expressed limited amount of secretory form IL-1Ra (sIL-1Ra) mRNA unless they were stimulated with LPS. The mRNA induction by LPS was further enhanced by 10 μM rolipram (*p* < 0.001), showing approximately 6.0-fold increase (Fig. 2B). The same experiments were also conducted with peritoneal macrophages using rolipram as well as the clinically approved PDE4 inhibitor roflumilast. The results indicated that both inhibitors also increased IL-1Ra release in a dose-dependent manner (Fig. 2C and E), and significantly enhanced sIL-1Ra mRNA expression (*p* < 0.001), albeit to a lesser extent compared with that in Raw 264.7 cells, showing approximately 2.2- and 2.7-fold increase with 10 μM rolipram and 1 μM roflumilast, respectively (Fig. 2D and F).

### PDE4 inhibitor does not alter intracellular form IL-1Ra mRNA level or cell viability in LPS-stimulated mouse peritoneal macrophages

To examine whether the lower sIL-1Ra mRNA induction by PDE4 inhibitors in peritoneal macrophages is due to a compensated increase in the intracellular form IL-1Ra (icIL-1Ra) mRNA expression, the icIL-1Ra mRNA levels in Raw 264.7 and mouse peritoneal macrophages were compared. The cells were treated with 10 ng/ml LPS for 3 h in the presence or absence of 10 μM rolipram. Real-time PCR results showed that, like sIL-1Ra mRNA induction, icIL-1Ra mRNA expression in Raw 264.7 cells also was greatly enhanced by rolipram, with approximately 6.8-fold increase (Supplementary Fig. 1A). However, no increase in icIL-1Ra mRNA was observed by PDE4 inhibition in peritoneal macrophages (Supplementary Fig. 1B), suggesting that the rolipram enhancement of IL-1Ra secretion in peritoneal macrophages is most likely derived from the associated sIL-1Ra mRNA induction.

As evidence indicates that icIL-1Ra can be released upon cell death^7^, we further probed the possibility that rolipram-enhanced IL-1Ra protein secretion in peritoneal macrophages may be contributed to some extent by the release of intracellular store of icIL-1Ra from an increased cell death. To this purpose, both macrophages were incubated with LPS in the presence or absence of rolipram for 8 h followed by the MTT assay. The results showed that with LPS alone the viability of Raw 264.7 cells was comparable to that of their control cells, whereas a decreased survival was detected in peritoneal macrophages (~85% survival, *P* = 0.06 compared with the control cells). However, we also observed that the viability of both macrophages was not reduced by rolipram (Supplementary Fig. 2), indicating that rolipram-enhanced IL-1Ra secretion in both macrophages has little or no contribution from the cell death-triggered icIL-1Ra release.

### Inhibition of PDE4 does not increase IL-1Ra mRNA stability

**Figure 3:**
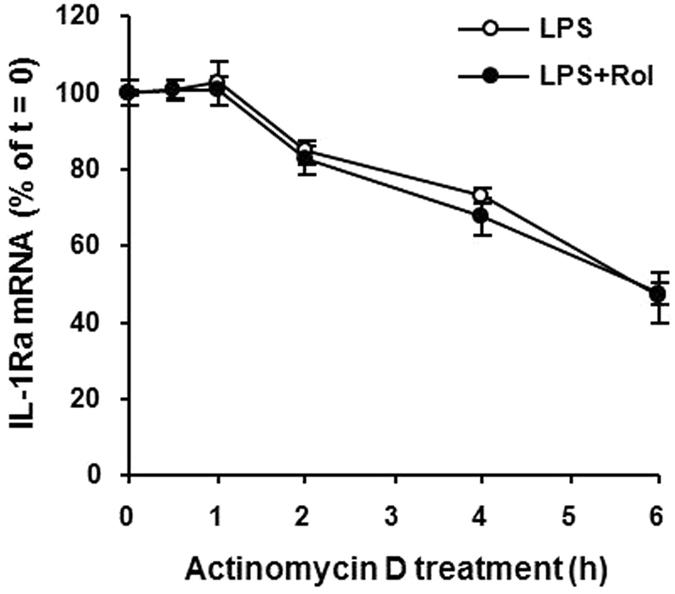
Rolipram does not alter sIL-1Ra mRNA stability in LPS-stimulated macrophages. Mouse peritoneal macrophages were treated with LPS (10 ng/ml) in the presence or absence of 10 μM rolipram for 3 h, followed by the addition of actinomycin D (10 μg/ml) to inhibit RNA transcription. The levels of sIL-1Ra mRNA were measured at 0, 0.5, 1, 2, 4 and 6 h after actinomycin D addition by real-time PCR. The amounts of sIL-1Ra mRNA were normalized to GAPDH mRNA levels. Values are expressed as percent of total sIL-1Ra mRNA at the time of actinomycin D addition. Data are the mean ± SEM (*n* = 4–6).

To investigate whether the steady-state level of sIL-1Ra mRNA induced by PDE4 inhibitors in macrophages is associated with an increase in transcription or mRNA stability, the RNA synthesis inhibitor actinomycin D was used to monitor the IL-1Ra mRNA expression profile in mouse peritoneal macrophages. The cells were stimulated with LPS in the presence or absence of rolipram for 3 h, followed by incubation with actinomycin D for different times. Real-time PCR analysis showed that the cells with or without rolipram treatment displayed a similar pattern of decrease in sIL-1Ra mRNA level up to at least 6 h (Fig. 3), indicating that the turnover rate or stability of this RNA was not affected by rolipram. Therefore, the rolipram-enhanced sIL-1Ra mRNA expression and protein secretion was mainly attributed to the increase in RNA transcription.

### Ablation of PDE4B increases LPS-induced IL-1Ra production in macrophages

**Figure 4:**
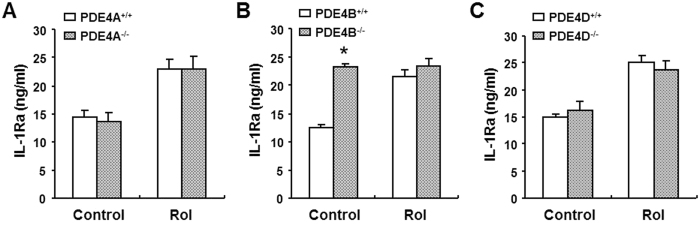
Effect of PDE4 inhibition on LPS-induced IL-1Ra production in PDE4-deficient macrophages. Peritoneal macrophages isolated from PDE4A^−/−^ (**A**), PDE4B^−/−^ (**B**) and PDE4D^−/−^ mice (**C**) and their corresponding wild-type mice were incubated with 10 μM rolipram for 20 min before LPS (10 ng/ml) stimulation for 8 h. IL-1Ra accumulation in the medium was determined by ELISA. Data are the mean ± SEM (*n* = 7–13 mice/group). *P < 0.001, compared with PDE4B^+/+^ control.

Rolipram is a non-selective PDE4 inhibitor which inhibits four PDE4 isoforms with similar potency. To date, no PDE4 inhibitors with isoform selectivity are available commercially. Among the four isoforms, three (PDE4A, 4B, and 4D) are expressed in mouse peritoneal macrophages^27^. To further dissect which PDE4 isoform is responsible for the pharmacological effect of rolipram on IL-1Ra production, PDE4 gene targeted mice were used. Peritoneal macrophages were isolated from PDE4A, 4B, and 4D null mice and their corresponding wild-type mice and incubated in the presence of LPS for 8 h. The IL-1Ra levels in the medium were measured by ELISA. As expected, the IL-1Ra release in all three wild-type macrophages was induced by LPS and this induction was enhanced by rolipram (Fig. 4). PDE4A^−/−^ and PDE4D^−/−^ macrophages also responded to LPS challenge as well as to rolipram induction in a manner similar to their wild-type counterparts (Fig. 4A and C). In contrast, LPS stimulation of PDE4B^−/−^ macrophages significantly increased IL-1Ra production compared with their wild-type cells (*p* < 0.001) (Fig. 4B). In addition, rolipram had no further effect on IL-1Ra production in PDE4B^−/−^ macrophages (Fig. 4B), indicating that the pharmacological inhibition of PDE4A and PDE4D in PDE4B^−/−^ macrophages had little or no effect on LPS-stimulated IL-1Ra production. These data demonstrate that rolipram enhances IL-1Ra response is mediated mostly, if not all, by inhibition of PDE4B but not PDE4A or PDE4D in macrophages.

In addition to peritoneal macrophages, bone marrow-derived macrophages (BMDM) were also prepared from PDE4B^+/+^ and PDE4B^−/−^ mice to test their IL-1Ra responses to LPS and rolipram. By quantitative PCR, we found that BMDM expressed relatively low level of PDE4A, 4B and 4D mRNA under a basal culture condition, while LPS markedly induced PDE4B, but not PDE4A or PDE4D, expression (Supplementary Fig. 3A), an observation consistent with the findings in human THP-1 cells and circulating monocytes^26^,^29^. Moreover, like in PDE4B^+/+^ and PDE4B^−/−^ peritoneal macrophages, LPS greatly induced IL-1Ra secretion in PDE4B^+/+^ BMDM, and this induction was further enhanced by rolipram. Furthermore, LPS stimulation of PDE4B^−/−^ BMDM significantly increased IL-1Ra secretion compared with their wild-type cells, whereas rolipram did not further increase the IL-1Ra response (Supplementary Fig. 3B). These results demonstrate that PDE4B also negatively regulate LPS-activated IL-1Ra responses in BMDM as in peritoneal macrophages.

### Cyclic AMP-PKA signaling mediates the stimulating effects of PDE4B ablation and rolipram inhibition on IL-1Ra production

**Figure 5:**
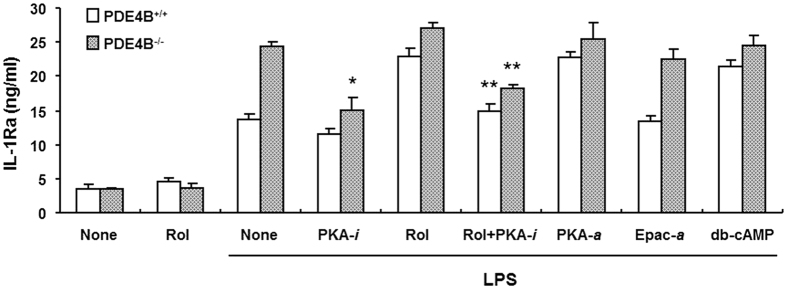
Effect of cAMP signaling on LPS-induced IL-1Ra production in mouse macrophages. Peritoneal macrophages from PDE4B^+/+^ and PDE4B^−/−^ mice were pretreated for 20 min with rolipram (10 μM), the PKA inhibitor Rp-8-CPT-cAMPS (PKA-*i*, 500 μM), the PKA activator 6-Bnz-cAMP (PKA-*α*, 250 μM), the Epac activator 8-pCPT-2′-O-Me-cAMP (Epac-*a*, 500 μM), the dibutyryl-cAMP (100 μM) or the combination of rolipram and PKA inhibitor, followed by LPS (10 ng/ml) stimulation for 8 h. IL-1Ra accumulation in the medium was measured by ELISA. Data are the mean ± SEM (*n* = 4–6). *P < 0.005, compared with PDE4B^−/−^ group treated with LPS alone. **P < 0.001, compared with the LPS + Rol group of the same genotype.

The cAMP regulation of cytokine production is known to be mediated by activation of either PKA or Epac^20^. To determine which signal pathway mediates the effect of PDE4 inhibitor or PDE4B ablation on IL-1Ra production, peritoneal macrophages isolated from PDE4B^+/+^ and PDE4B^−/−^ mice were incubated with LPS in the presence or absence of different cAMP analogs for 8 h. Figure 5 shows that IL-1Ra production in LPS-stimulated PDE4B^+/+^ cells was significantly induced by the PKA activator 6-Bnz-cAMP as well as the cAMP analog dibutyryl-cAMP (db-cAMP), and the levels of induction were similar to those in rolipram-treated cells. Contrarily, the Epac activator 8-pCPT-2′-O-Me-cAMP had no significant effect on IL-1Ra response in these cells. Additionally, db-cAMP and PKA activator, like rolipram, exhibited little or no further increase in IL-1Ra production in PDE4B^−/−^ macrophages compared with these cells treated with LPS alone. These results demonstrate that the enhancement of IL-1Ra production by either PDE4 inhibition or PDE4B ablation is cAMP-dependent and is mediated by cAMP-PKA but not cAMP-Epac signal pathway.

More importantly, using the PKA inhibitor Rp-8-CPT-cAMPS to treat these macrophages, we found that IL-1Ra accumulation was not significantly altered in LPS-stimulated PDE4B^+/+^ cells (Fig. 5), indicating that the IL-1Ra response to LPS alone is independent of PKA activity. Conversely, treatment of PDE4B^−/−^ macrophages with the PKA inhibitor caused a significant decrease in IL-1Ra production to levels not significantly different from those in PDE4B^+/+^ cells treated with LPS alone. Moreover, when the PDE4B^+/+^ cells were incubated with a combination of rolipram and the PKA inhibitor, the IL-1Ra induced by rolipram also was reduced significantly (P < 0.001) to the level similar to that in PDE4B^+/+^ cells treated with LPS alone. These data demonstrate that the upregulation of IL-1Ra by PDE4B inactivation requires an active PKA (Fig. 5).

### LPS-induced IL-1Ra production in PDE4B null macrophages requires both PKA-dependent and PKA-independent CREB activation

**Figure 6:**
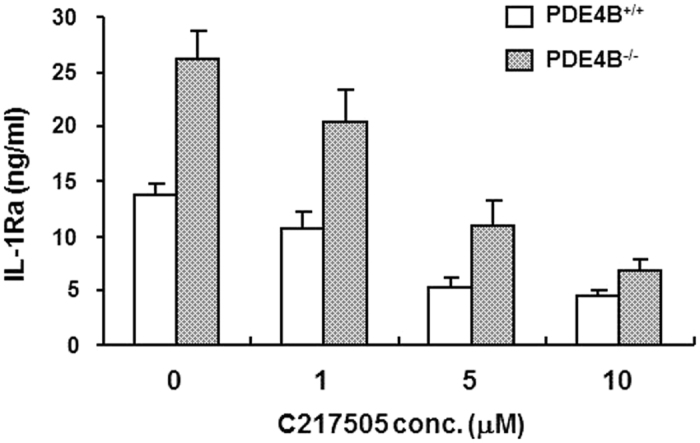
The CREB activity mediates LPS-induced IL-1Ra production in PDE4B^+/+^ and PDE4B^−/−^ macrophages. Peritoneal macrophages from PDE4B^+/+^ and PDE4B^−/−^ mice were incubated with increasing concentrations of the CREB inhibitor 217505 for 20 min before LPS (10 ng/ml) stimulation for 8 h. Accumulation of IL-1Ra in the medium was measured by ELISA. Data are the mean ± SEM (*n* = 6).

It is generally accepted that gene transcription induced by cAMP-PKA signaling is largely mediated via activation of cAMP response element-binding protein (CREB). As mentioned above, in wild-type macrophages IL-1Ra production elicited by LPS alone is not mediated by PKA activation, whereas the rolipram-enhanced response is mostly dependent on PKA activity. To further determine whether such PKA-mediated IL-1Ra expression is regulated by the PKA-CREB axis, the CREB inhibitor C217505, a CREB-binding protein (CBP)-CREB interaction inhibitor, was used and the IL-1Ra release in PDE4B^+/+^ and PDE4B^−/−^ macrophages was compared. As shown in Fig. 6, by preincubation of the cells with increasing concentrations of C217505 for 20 min followed by 8 h LPS stimulation, a dose-dependent decline of IL-1Ra production was observed in both cells. At 10 μM of C217505, the levels of IL-1Ra release in PDE4B^−/−^ cells were not significantly different from those observed in PDE4B^+/+^ cells (Fig. 6), indicating that IL-1Ra production provoked by TLR4 activation in both wild-type and PDE4B null macrophages is mediated mostly by CREB activation. However, the activation of CREB in LPS-stimulated wild-type cells is independent of PKA activity as IL-1Ra response to LPS alone was not blocked by PKA inhibitor in these cells (Fig. 5). Conversely, the CREB activation triggered by PDE4B ablation to enhance IL-1Ra production is mostly dependent on PKA activity. Taken together, these results demonstrate that the IL-1Ra response to LPS in PDE4B null macrophages is controlled by both PKA-dependent and PKA-independent CREB activation.

### STAT3 activation elicited by PDE4B ablation is not fully responsible for the observed IL-1Ra upregulation

**Figure 7:**
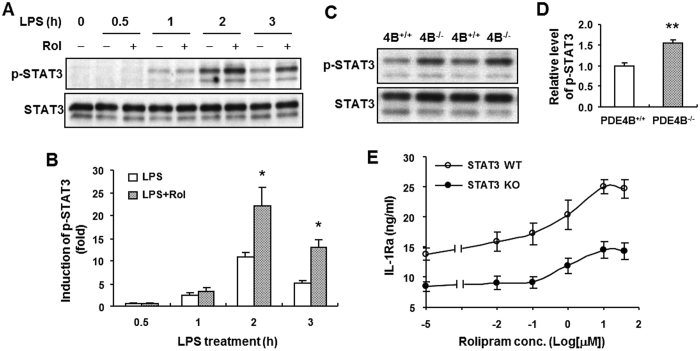
STAT3 phosphorylation enhanced by rolipram or PDE4B ablation does not contribute to the increased IL-1Ra production in LPS-stimulated macrophages. Peritoneal macrophages from PDE4B^+/+^ mice were incubated with LPS (100 ng/ml) in the presence or absence of 10 μM rolipram for indicated times (**A**), or the cells from both PDE4B^+/+^ and PDE4B^−/−^ mice were treated with LPS (100 ng/ml) for 2 h (**C**). The STAT3 phosphorylation (Tyr^705^) was detected by immunoblotting as described in Methods (**A** and **C**). Representative Western blots are shown. The blots were cropped for improving clarity and full-length blots are presented in Supplementary Figs 4 and 5. The level of phosphorylation relative to total STAT3 protein was quantified and expressed as fold induction to unstimulated control (**B**) or to the PDE4B^+/+^ group (**D**). *P < 0.05, compared with the corresponding groups treated with LPS alone (*n* = 4 in B); **P < 0.005, compared with the PDE4B^+/+^ group (*n* = 3 in **D**). (**E**) Bone marrow-derived macrophages prepared from STAT3^+/+^ and STAT3^−/−^ mice were incubated with increasing concentrations of rolipram for 20 min prior to LPS (100 ng/ml) stimulation for 8 h. IL-1Ra accumulation in the medium was measured by ELISA. Data are the mean ± SEM (*n* = 7–8).

Stimulation of macrophages with LPS has been shown to induce phosphorylation of signal transducer and activator of transcription (STAT)3 at Tyr^705^, and this STAT3 activation is enhanced by the presence of the cAMP elevator prostaglandin E_2_ (PGE_2_)^30^. Additionally, a previous study by Carl *et al*. demonstrated that the IL-10-induced IL-1Ra expression in LPS-stimulated macrophages is mediated by an increase in STAT3-Tyr^705^ phosphorylation and transcription^14^. These findings prompted us to investigate whether elevation of cAMP by PDE4 inhibitor or PDE4B ablation promotes STAT3 phosphorylation, and whether such phosphorylation mediates the enhanced IL-1Ra production in LPS-stimulated PDE4B^−/−^ macrophages. For this purpose, we first treated wild-type peritoneal macrophages with LPS in the presence or absence of rolipram for various times. Western blotting using an antibody specific to Tyr^705^-phospho-STAT3 revealed that little or no STAT3 phosphorylation was detected in the untreated cells (Fig. 7A and B). The protein phosphorylation was increased with time up to 2 h and declined thereafter. The level of phosphorylation was significantly enhanced when the cells were incubated with rolipram (*p* < 0.05), reaching approximately 2.0- and 2.6-fold increases at 2 h and 3 h LPS stimulation, respectively (Fig. 7A and B). We further probed the effect of PDE4B on STAT3 phosphorylation and found that following 2 h LPS treatment the level of STAT3 Tyr^705^ phosphorylation was significantly increased in 4B^−/−^ macrophages compared with the wild-type cells (P < 0.005) (Fig. 7C and D). These results support the notion that STAT3 activation can be induced by cAMP-elevating agents in LPS-stimulated macrophages.

To further assess whether the STAT3 phosphorylation enhanced by rolipram and PDE4B ablation is associated with the observed IL-1Ra induction, bone marrow-derived macrophages (BMDM) from STAT3 null mice and the corresponding wild-type mice were prepared, and their IL-1Ra production in response to LPS and rolipram was compared. The reason that BMDM instead of peritoneal macrophages were used in this study was that we needed enough cells to conduct the IL-1Ra response experiment as proposed as well as Western blotting to confirm that the STAT3 deletion was fully induced in BMDM from each STAT3 targeted mouse. The number of peritoneal macrophage isolated from each mouse was not enough for this purpose. As shown in Fig. 7E, after 8 h LPS stimulation in the absence or low concentrations of rolipram IL-1Ra accumulation was significantly decreased in STAT3^−/−^ cells compared with STAT3^+/+^ cells (P < 0.001), indicating that STAT3 is essential for LPS-induced IL-1Ra production. Consistent with the results observed in peritoneal macrophages, LPS-stimulated IL-1Ra accumulation in wild-type BMDM was enhanced with increasing concentrations of rolipram, showing approximately 1.81-fold increase compared with the cells treated with LPS alone. However, a similar level of IL-1Ra induction by rolipram (approximately 1.73 folds) also was observed in the STAT3^−/−^ BMDM (Fig. 7E), indicating that this rolipram effect was not caused by STAT3 activation because no STAT3 phosphorylation exists in these cells. However, by comparing the absolute amounts of IL-1Ra release between the treatment conditions of LPS alone and LPS + Rol, we found that IL-1Ra secretion induced by 10 μM rolipram was greater in STAT3^+/+^ cells (~11.2 ng/ml) compared with STAT3^−/−^ cells (~6.1 ng/ml) (Fig. 7E), indicating that the rolipram-induced IL-1Ra production can be triggered by both STAT3-independent (as detected in STAT3^−/−^ cells) and STAT3-dependnet pathways in STAT3^+/+^ cells. Taken together, these data suggest that the rolipram- or PDE4B ablation-enhanced STAT3 phosphorylation is not fully responsible for the enhanced IL-1Ra production in PDE4B^−/−^ macrophages or rolipram-treated PDE4B^+/+^ macrophages.

### Serum level of IL-1Ra is elevated in PDE4B-deficient mice during LPS-induced sepsis

**Figure 8:**
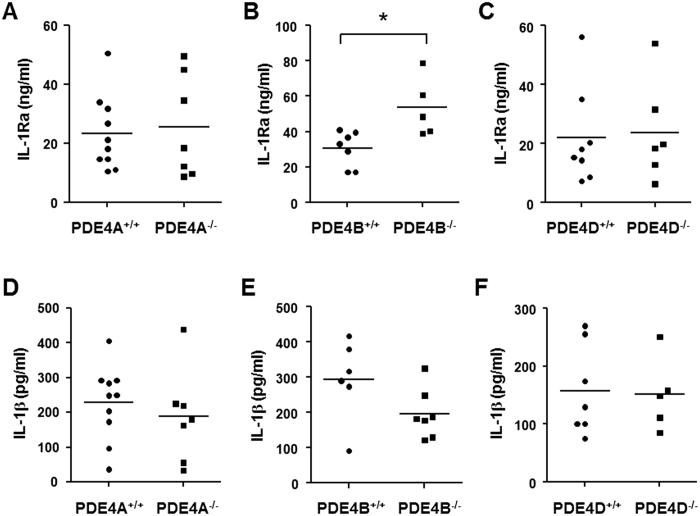
Serum levels of IL-1Ra and IL-1β in PDE4 null mice after high-dose LPS challenge. PDE4A^−/−^ (**A** and **D**), PDE4B^−/−^ (**B** and **E**) and PDE4D^−/−^ mice (**C** and **F**) and their corresponding wild-type mice were injected intraperitoneally with 10 mg/kg LPS. After 6 h, blood samples were collected by heart puncture and the serum levels of IL-1Ra (**A**–**C**) and IL-1β (**D**–**F**) were measured by ELISA. The result of individual mouse is plotted as a symbol and the mean of each group expressed as a line. *P < 0.05 (*n* = 5–10 mice/group).

Overexpression of IL-1Ra has been shown to protect mice from the lethal effects of endotoxin^31^. Additionally, gram-negative bacteria-induced septic shock is associated with an elevation of plasma IL-1Ra in patients, a response believed to be an attempt to suppress the shock syndrome^32^. These reports, together with our previous observation that ablation of PDE4B protects mice from LPS-induced shock^27^, prompted us to assess the serum levels of IL-1Ra in PDE4B null mice following LPS provocation. To this purpose, PDE4 null mice and their corresponding wild-type mice were challenged with a high dose of LPS (250 μg/25 g body weight), and after 6 h blood samples were collected. The serum IL-1Ra was undetectable in mice without LPS treatment. Figure 8A–C shows that the serum level of IL-1Ra was significantly higher (by 75%) in PDE4B^−/−^ mice compared with the PDE4B^+/+^ mice (P < 0.05), whereas no significant difference was observed between PDE4A^−/−^ or PDE4D^−/−^ mice and their corresponding wild-type mice. These data indicate that ablation of PDE4B, but not PDE4A or PDE4D, alters TLR signaling *in vivo*, leading to an increase in circulating IL-1Ra.

In addition to the measurement of IL-1Ra, the serum levels of TNF-α and IL-1β, two key proinflammatory cytokines involved in the LPS-sepsis, were also quantified. The ELISA results showed that IL-1β levels were not changed in PDE4A^−/−^ or PDE4D^−/−^ mice, while a trend of decrease of this cytokine was observed in PDE4B^−/−^ mice (*P* = 0.10), suggesting a downregulation of IL-1β might play a role in the protection of PDE4B^−/−^ mice from LPS-induced shock (Fig. 8D–F). Regarding the TNF-α level, the results indicated that most of the serum samples were under the detection limit of the assay. The possible explanations are that TNF-α is expressed as an early cytokine and has a relatively short half-life in serum^33^.

## Discussion

The PDE4 inhibitor rolipram has been shown to increase IL-1Ra secretion in Raw 264.7 macrophages in response to LPS^21^, but the molecular mechanism underlying this effect remained to be elucidated. In the present study, we show that the IL-1Ra upregulation by PDE4 inhibitor is mediated by inhibition of PDE4B, one of the three PDE4 isoforms expressed in macrophages, demonstrating that, in addition to pro-inflammatory cytokines, PDE4B is also involved in the gene regulation of anti-inflammatory cytokine. Additionally, we also found that IL-1Ra production initiated by LPS alone is PKA independent, whereas it requires CREB activation. The IL-1Ra response enhanced by PDE4B ablation or PDE4 inhibition, however, depends on both PKA and CREB in LPS-stimulated macrophages, and this activated cAMP-PKA-CREB signaling can cross talk with LPS-TLR4 signal pathway, directly or indirectly, to promote IL-1Ra transcription.

Using the PDE4 gene targeting mice, here we demonstrate that ablation of PDE4B, but not PDE4A or PDE4D, enhances the LPS-induced IL-1Ra production, and the level of increase is similar to that induced by rolipram or db-cAMP in LPS-treated wild-type macrophages (Fig. 5). These results indicate that the upregulation of IL-1Ra by PDE4B ablation is elicited by cAMP elevation in these cells. This is supported by the findings of Feng *et al*. that 8-bromo-cAMP and cholera toxin, both cAMP elevators, positively regulate IL-1Ra mRNA expression in LPS-stimulated macrophages^19^.

As reported by other investigators, we also observed that sIL-1Ra gene expression and secretion are upregulated in macrophages in response to the endotoxin LPS^5^,^34^. This IL-1Ra production initiated by LPS alone appears to be regulated by different signaling molecules. A study by Molnarfi *et al*. has demonstrated that inhibition of PI3K activity suppresses IL-1Ra production in LPS-stimulated human monocytes^16^. A following study by Rehani *et al*. further revealed that the downstream component glycogen-synthase kinase 3 (GSK3) in the PI3K-Akt pathway negatively regulates the LPS-induced IL-1Ra levels due to its ability to modulate ERK1/2 activity^18^. In this study, we showed that the IL-1Ra production in macrophages induced by LPS was not PKA dependent because the IL-1Ra induction was not reversed by the treatment with the PKA inhibitor Rp-8-CPT-cAMPS. This result may be explained by the fact that the expression and activity of PDE4B is induced by LPS in macrophages^27^. It is known that induction of PDE4 isoforms, including PDE4B, in a cell is able to reduce intracellular cAMP and subsequently inactivates PKA. As a result, the observed increase in IL-1Ra production in response to LPS most likely is PKA independent. Moreover, our data indicated that this IL-1Ra regulation by LPS requires CREB activation, as CREB inhibitor dose-dependently blocks the IL-1Ra production (Fig. 6). This is in agreement with the findings of Avni *et al*.^35^, showing that LPS stimulates CREB phosphorylation on Ser-133 in Raw 264.7 macrophages and this process is mediated by mitogen- and stress-activated kinase 1 (MSK1), but not PKA. Additionally, using the MSK-deficient macrophages, Darragh *et al*. have demonstrated that MSK1 and 2 activated by either ERK1/2 or p38 are essential for LPS-induced IL-1Ra production^17^. Taken together, these studies indicate that the mechanism involved in the induction of IL-1Ra by LPS alone is distinct from that initiated by PDE4B inactivation.

Cyclic AMP-elevating agents, including PDE4 inhibitors, are known to regulate a wide range of inflammatory processes in macrophages, predominantly via the activation of the effector PKA or Epac^20^,^28^. The role of PKA and Epac in regulation of cytokine and chemokine production varies by individual responses and cell types^36^,^37^,^38^. Activation of cAMP signaling in macrophages has been shown to suppress the production of several pro-inflammatory mediators, such as LPS-induced TNF-α and macrophage inflammatory protein-1α (MIP-1α or CCL3)^36^, and ionophore A23187-stimulated leukotriene B4^39^, as well as to enhance LPS-induced production of the anti-inflammatory cytokine IL-10^36^. The regulation of all these responses depends on PKA but not Epac. However, Epac has been implicated in the suppression of LPS-induced interferon-β expression in J774A.1 macrophages^40^ as well as the induction of the expression of several pro-inflammatory chemokines, such as CXCL5, CXCL7, and CCL2 in human monocyte-derived macrophages^41^. Our present data demonstrate that the upregulation of IL-1Ra, like our previously observed downregulation of TNF-α and CCL3 21,27, by PDE4B ablation or rolipram inhibition in LPS-stimulated macrophages is PKA dependent and Epac independent. The two opposite transcriptional effects resulted from the same cAMP-PKA activation suggest that distinct transcriptional machineries are prompted by PKA in PDE4B-deficient macrophages, all aiming to reduce endotoxin-induced inflammation. The exact PKA downstream signaling pathways and transcriptional components involved in the expression of anti-inflammatory (IL-1Ra) and pro-inflammatory cytokines (TNF-α and CCL3) remain to be identified.

Using wild-type and STAT3-deficient BMDM, we show here that STAT3 activity is essential for LPS-induced IL-1Ra secretion (Fig. 7E), confirming the role of STAT3 as a positive regulator in IL-1Ra production under endotoxin stimulation. The LPS-induced STAT3 phosphorylation has been shown to require endogenously produced IL-10, an anti-inflammatory cytokine also induced in response to LPS stimulation, and this STAT3 activation results in the induced IL-1Ra expression in BMDM^14^. In addition to the induction by LPS alone, STAT3 phosphorylation also was enhanced by rolipram or PDE4B ablation in LPS-stimulated macrophages (Fig. 7). This increase in STAT3 activity, however, did not contribute entirely to the enhanced IL-1Ra production observed in the PDE4B^−/−^ macrophages or rolipram-treated PDE4B^+/+^ cells simply because an increase in IL-1Ra production initiated by rolipram was still detected in macrophages deficient in STAT3. These results suggest that STAT3 phosphorylation induced by PDE4B inactivation may play a role in regulating not yet identified cellular processes in LPS-stimulated macrophages.

IL-1Ra is a naturally occurring inhibitor of the pro-inflammatory cytokine IL-1. Our observation that inactivation of PDE4B upregulates this anti-inflammatory cytokine supports the view that PDE4 inhibitors with PDE4B selectivity functions as negative modulators concerning inflammation^28^,^42^. PDE4B deficiency has been demonstrated to attenuate a number of inflammatory cell responses and inflammatory disorders in animal models. As described above, the production of the pro-inflammatory cytokine TNF-α and chemokine CCL3 is markedly reduced in PDE4B null macrophages in response to LPS^21^,^26^,^27^. In an allergic asthma model, PDE4B ablation impairs allergen-specific T-cell proliferation and Th2 cell functions as well as protects the animal from allergen-induced airway hyperresponsiveness^43^. Moreover, PDE4B knockdown with interference RNA has also been shown to attenuate lung inflammation in LPS-induced acute lung injury^44^, as well as spinal nerve ligation (SNL)-induced neuropathic pain and inflammatory responses in the spinal cord^45^. Given the tolerability concerns with non-selective PDE4 inhibitors, including the clinically used roflumilast and apremilast^28^, these findings provide insights into the pharmacological potential of PDE4B-selective inhibitors.

By binding to IL-1 receptor on cell surface, IL-1Ra prevents IL-1 from sending a signal to that cell and thereby counteracting the action of IL-1. Deficiency of IL-1Ra is implicated in life-threatening systemic inflammation with skin and bone involvement^11^,^12^. Treatment of these patients with the human recombinant IL-1Ra anakinra resolves the symptoms and lesions rapidly. A number of acute and chronic inflammatory conditions in which IL-1 plays a major role are demonstrated to respond to IL-1 blockers, such as anakinra and anti-IL-1β monoclonal antibodies^46^. Anakinra is approved for the treatment of the autoimmune disease rheumatoid arthritis (RA). In RA patients the production of endogenous IL-1Ra in synovium and synovial fluid appears to be insufficient to balance the elevated IL-1 levels^1^. In this study, we found that the serum level of IL-1Ra was significantly increased in mice after a high dose of LPS challenge, and it was approximately 75% higher in PDE4B^−/−^ mice compared with PDE4B^+/+^ mice. Moreover, the level of IL-1β was lower in PDE4B^−/−^ mice than in PDE4B^+/+^ mice (P = 0.10). These results infer that PDE4B-selective inhibitors, developed as non-peptide anti-inflammatory agents, may be used as alternatives of anakinra for the treatment of IL-1-driven diseases.

In summary, our data demonstrate that inhibition of PDE4B, one of the three PDE4 isoforms expressed in macrophages, upregulates anti-inflammatory cytokine IL-1Ra production in endotoxin-stimulated macrophages *in vitro* and sepsis *in vivo*. These results, together with previous findings that PDE4B ablation suppresses pro-inflammatory cytokine responses, provide proof of concept that PDE4B-selective inhibitors may retain the anti-inflammatory effects of non-selective PDE4 inhibitors.

## Methods

### Reagents

Dibutyryl-cAMP, rolipram, roflumilast, *Escherichia coli* LPS (055:B5), goat γ-globulin, 3-(4,5-Dimethyl-2-thiazolyl)-2,5-diphenyl-2H-tetrazolium bromide (MTT) and actinomycin D were obtained from Sigma-Aldrich (MO, USA), and the CREB inhibitor C217505 was from Merck Millipore (MA, USA). The PKA activator N^6^-benzoyladenosine-3′, 5′-cyclic monophosphate (6-Bnz-cAMP), Epac activator 2′-O-methyladenosine-3′, 5′-cyclic monophosphate (8-pCPT-2′-O-Me-cAMP), and PKA inhibitor 8-(4- Chlorophenylthio)-8-(4-Chlorophenylthio) adenosine-3′, 5′-cyclic monophosphorothioate, Rp-isomer (Rp-8-CPT-cAMPS) were purchased from BioLog Life Science Institute (Bremen, Germany). The RPMI 1640 and DMEM medium base and fetal bovine serum (FBS) were obtained from Thermo Fisher Scientific (MA, USA).

### Cell line

Raw 264.7, a murine leukemia macrophage cell line, was obtained from Bioresource Collection and Research Center (Taiwan). The cells were maintained in DMEM medium supplemented with 100 U/ml penicillin, 100 μg/ml streptomycin, and 10% FBS at 37 °C in 5% CO_2_. To assess the effects of LPS and rolipram on IL-1Ra release, Raw 264.7 cells were plated at 2.5 × 10^5^ cell/ml in 96-well plate, grown overnight, and then incubated with drugs for desired times. The culture medium supernatant was collected for IL-1Ra ELISA. For quantitative PCR analysis on secretory IL-1Ra (sIL-1Ra) and intracellular IL-1Ra (icIL-1Ra) mRNA expression, the cells were incubated with LPS and rolipram for 3 h in the same medium.

### Mice

Generation of PDE4A-, PDE4B- and PDE4D-deficient mice has been described previously^21^. The PDE4A and PDE4B null mice have been backcrossed from the mixed 129/Ola × C57Bl/6 to the pure C57BL/6 background for at least 12 generations, whereas the PDE4D null mice were kept in the mixed background because these mice become embryonic lethal when the null allele is transferred to the pure C57Bl/6 background. Generation of MxCre-STAT3^f/f^ mice with a conditional *STAT3* allele and induction of the STAT3 deletion have been described previously^47^,^48^. Mice were bred and maintained at 23 °C with a 12-hour light/12-hour dark cycle and provided with food and water *ad libitum* in the animal facility at National Center University. Mice used in this study were 2–5 months of age. All the experimental procedures involving animals were approved by the Institutional Animal care and Use Committee (IACUC) at the authors’ institutes. Animal care and experimental procedures were carried out in accordance with the guidelines of the IACUC of authors’ institutes.

### Primary macrophages

Peritoneal macrophages were isolated and purified from PDE4-deficient mice and their corresponding wild-type mice, and cultured in RPMI 1640 medium supplemented with 100 U/ml penicillin, 100 μg/ml streptomycin, and 10% FBS at 37 °C in 5% CO_2_ as described previously^27^. Briefly, cells were collected by washing the peritoneal cavity with cold HBSS (Thermo Fisher Scientific) followed by centrifugation, and the cell pellet was resuspended in PBS containing 2% heat-inactivated FBS. To deplete B cells, the peritoneal cells were plated in a 10-cm Petri dish that was pretreated with 35 μg of goat anti-mouse IgG + IgM (H + L) (Jackson ImmunoReserach Laboratories) and 300 μg of goat γ-globulin in 10 ml of PBS. After incubation at 4 °C for 1 h, non-adherent cells enriched with macrophages were harvested and centrifuged, and the cell pellet was resuspended in the RPMI1640 medium. Macrophages were counted and plated at 4.5 × 10^5^/ml in 96-well plate or 1 × 10^6^/ml in 3.5-cm plate.

Bone marrow–derived macrophages (BMDM) were established as previously described^49^. Briefly, bone marrow cells were flushed from mouse femurs and tibias with PBS, followed by RBC lysis, PBS wash, and then plating at a density of 5 × 10^5^ cell/ml in 10-cm petri dish. The cells were cultured in RPMI 1640 medium containing 20% L929 cell-conditioned medium, 10% FBS, 100 U/ml penicillin, and 100 μg/ml streptomycin at 37 °C in 5% CO_2_ for 5–7 days, in which 5 ml of the same medium was added on day 3. Before use, the cells were scrapped off the original petri dish and plated at 0.8 × 10^6^ cell/ml in 6-well plate or 4.5 × 10^5^ cell/ml in 96-well plate in the same RPMI 1640 medium.

The plated peritoneal macrophages and BMDM were cultured overnight, and then incubated with LPS in the absence or presence of rolipram, roflumilast, cAMP analogs, or other test drugs. The medium supernatants were collected for ELISA and the cells for immunoblotting or quantitative PCR analysis.

### RNA isolation, cDNA synthesis, and quantitative PCR

Total RNA was extracted from Raw 264.7, mouse peritoneal macrophages, and BMDM with the TRIzol reagent (Thermo Fisher Scientific, MA, USA) according to the manufacturer’s protocol. First strand cDNA was synthesized from 0.5 to 1 μg of total RNA in the presence of random primer using M-MLV reverse transcriptase according to the manufacturer’s instructions (Thermo Fisher Scientific, MA, USA). Real-time PCR was carried out with the SYBR FAST qPCR Master Mix (KAPA Biosystems, MA, USA) using iQ5 Real-time PCR Detection System (Bio-Rad, CA, USA). The reaction was performed in a 20 μl reaction mixture with preliminary denature for 3 min at 94 °C, followed by 40 cycles of denaturing at 94 °C for 30 sec, annealing at 60 °C for 45 sec, and extension at 72 °C for 30 sec. Oligonucleotide primer sequences were as follows: sIL-1Ra, 5′-AGTCGCTAGTCTCTATTGCC-3′ and 5′-TTCTGAAGGCTTGCATCTTG-3′; icIL-1Ra, 5′-TCCTTTATACACAGCAAGTCTC-3′ and 5′-TTCTGAAGGCTTGCATCTTG-3′; PDE4A, 5′-CTTCTGCGAGACCTGCTCCA-3′ and 5′-GAGTTCCCGGTTCAGCATCC-3′; PDE4B, 5′-GCCACTGGATGAGAGGAGCA-3′ and 5′-CCTTTTCCGGTCCCTCAGAA-3′; PDE4D, 5′-ACCGCCAGTGGACGGACCGGA-3′ and 5′-CATGCCACGCTCCCGCTCTCGG-3′; GAPDH, 5′-GGAGCGAGACCCCACTAACA-3′ and 5′-ACATACTCAGCACCGGCCTC-3′. All primers used were synthesized by Tri-I Biotech (Taiwan). Target gene expression was calculated by the comparative ΔΔ cycle threshold (C_t_) method for relative quantification after normalization to the housekeeping gene *GAPDH* expression.

### RNA stability assay

Mouse peritoneal macrophages were treated with LPS (10 ng/ml) in the presence or absence of 10 μM rolipram for 3 h. The cells then were incubated with actinomycin D (10 μg/ml) for 0, 0.5, 1, 2, 4 and 6 h. Total RNA was extracted from the cells and sIL-1Ra mRNA levels were measured by quantitative PCR.

### Western blot analysis

Following treatment with rolipram and LPS, macrophages were washed once with cold PBS, gently scraped off the culture dish in a lysis buffer (50 mM Tris-Cl, pH 7.5, 250 mM sodium chloride, 5% glycerol, 10 mM sodium fluoride, 1 mM EDTA, 0.2 mM EGTA, 10 mM sodium pyrophosphate, 1 mM sodium orthovanadate, 1 mM 4-(2-aminoethyl)-benzenesulfonyl fluoride (Roche, CA, USA), 1 tablet/10 ml of protease inhibitor mixture (Roche, CA, USA), 0.4% Nonidet P-40, and 14.3 mM 2-mercaptomethanol), and then incubated on ice for 20 min. After sonication with 25 bursts, the cell lysate was centrifuged at 4 °C for 15 min at 16,000 × g. Aliquots of the supernatant were subjected to electrophoresis on 10% SDS-PAGE, and then blotted onto a polyvinylidene fluoride (PVDF) membrane (Millipore, MA, USA). Phosphorylated STAT3 was detected by incubation of the membrane with an anti- phospho-STAT3 (Tyr705) mAb (1:2000; Cell Signaling Technology, MA, USA) followed by a peroxidase-conjugated goat anti-mouse secondary Ab (1:5000; Jackson ImmunoResearch, PA, USA). Immunoreactive signals were visualized using an ECL detection system (Perkin Elmer, MA, USA). To detect total STAT3 protein, the stripped membrane was incubated with a STAT3 Ab (1:2000; BD Biosciences, CA, USA). Signals of the immunoreactive bands were quantified using GelPro31 software, and all phosphorylated signal intensities were normalized with their total proteins.

### LPS-induced systemic inflammation

PDE4A, 4B, and 4D null mice and their corresponding wild-type mice were injected with LPS (10 mg/kg body weight) intraperitoneally to induce systemic inflammation. After 6 h, mouse blood was collected by cardiac puncture and serum obtained from blood clotting. The serum levels of IL-1Ra, IL-1β and TNF-α were measured by ELISA.

### Cytokine measurement

Levels of IL-1Ra, IL-1β and TNF-α in macrophage culture supernatants and blood sera were measured with commercially available enzyme-linked immunosorbent assay (ELISA) kits (IL-1Ra from R&D Systems, MN, USA; IL-1β and TNF-α from BD Biosciences, CA, USA). The sensitivities of the assay were 156.25 pg/ml, 31.3 pg/ml, and 15.6 pg/ml, respectively.

### Cell viability assay

The cell viability was determined by the MTT assay. Raw 264.7 and peritoneal macrophages were plated in 96-well plate at 1.25 × 10^5^ and 4.5 × 10^5^ cell/ml, respectively. Following overnight incubation, cells were treated with LPS in the presence or absence of rolipram for 8 h. The MTT solution then was added to each well at a final concentration of 0.5 mg/ml. After 3 h incubation, the medium was removed and 200 μl DMSO was added to each well to dissolve the formed formazan. Then the absorbance was determined using a microplate reader (VersaMax, Molecular Devices, CA, USA) at the wavelength of 570 nm. The cell viability was expressed as percent survival of untreated cells.

### Statistical analysis

All data are expressed as the mean ± SEM from at least three independent experiments. Differences between different treatment groups were assessed using the Student’s t-test. Values of *P* < 0.05 were considered statistically significant.

## Additional Information

**How to cite this article:** Yang, J.-X. *et al*. Phosphodiesterase 4B negatively regulates endotoxin-activated interleukin-1 receptor antagonist responses in macrophages. *Sci. Rep.* **7**, 46165; doi: 10.1038/srep46165 (2017).

**Publisher's note:** Springer Nature remains neutral with regard to jurisdictional claims in published maps and institutional affiliations.

## Supplementary Material

Supplementary Figures
