# Supplementary material for: Phosphodiesterase 4B negatively regulates endotoxin-activated interleukin-1 receptor antagonist responses in macrophages
Source: Sci Rep. 2017 Apr 6;7:46165. doi: 10.1038/srep46165 (PMC5382768; doi:10.1038/srep46165)
Supplement: Supplementary Figures [file srep46165-s1.pdf]

**Phosphodiesterase 4B negatively regulates endotoxin-activated interleukin-1 receptor antagonist responses in macrophages**

Jing-Xing Yang[1], Kou-Chou Hsieh[2], Yi-Ling Chen[1], Chien-Kuo Lee[3], Marco Conti[4], Tsung-Hsien Chuang[5], Chin-Pyng Wu[2,*] and S.-L. Catherine Jin[1,*]

[1]Department of Life Sciences, National Central University, Zhongli District, Taoyuan City, Taiwan, Republic of China.

[2]Internal Medicine, Landseed Hospital, Pingzhen District, Taoyuan City, Taiwan, Republic of China.

[3]Graduate Institute of Immunology, National Taiwan University College of Medicine, Taipei, Taiwan, Republic of China.

[4]Department of Obstetrics, Gynecology, and Reproductive Sciences, University of California San Francisco, California, USA.

[5]Immunology Research Center, National Health Research Institutes, Miaoli, Taiwan, Republic of China.

*These authors contributed equally to this work.

***Correspondence:** Professor S.-L. Catherine Jin, Department of Life Sciences, National Central University, 300 Zhongda Road, Zhongli District, Taoyuan City, Taiwan, R.O.C. Tel: +886-3-4227151 ext. 65066, e-mail: slcj@ncu.edu.tw

## SUPPLEMENTARY FIGURE LEGENDS

**sFigure 1. Effect of rolipram on LPS-induced icIL-1Ra mRNA expression in mouse macrophages.** Raw 264.7 cells (A) and mouse peritoneal macrophages (B) were pretreated for 20 min with 10 µM rolipram prior to LPS (10 ng/ml) stimulation for 3 h. The icIL-1Ra mRNA levels in the cells were determined by real-time PCR and expressed as fold induction to the untreated cells. Data are the mean ± SEM (*n* = 4 in A; *n* = 5 in B).

**sFigure 2. Rolipram does not alter cell viability in LPS-stimulated macrophages.** Raw 264.7 cells (A) and mouse peritoneal macrophages (B) were pretreated for 20 min with 10 µM rolipram before LPS (10 ng/ml) stimulation for 8 h. Cell viability was determined by the MTT assay as described in the Methods. Data are the mean ± SEM (*n* = 4 in A; *n* = 5-6 in B).

**sFigure 3. PDE4 isoform expression and regulation of PDE4B ablation on IL-1Ra secretion in LPS-stimulated bone marrow-derived macrophages.** (A-C) Bone marrow-derived macrophages (BMDM) were incubated with or without LPS (100 ng/ml) for 3 h. The levels of PDE4A (A), PDE4B (B), and PDE4D (C) mRNA were determined by real-time PCR and expressed as fold change to the untreated cells. (D) BMDM prepared from PDE4B$^{+/+}$ and PDE4B$^{-/-}$ mice were pretreated for 20 min with rolipram (10 µM) followed by LPS (100 ng/ml) stimulation for 8 h. IL-1Ra

accumulation in the medium supernatant was measured by ELISA. Data are the mean ± SEM (*n* = 5 in A-C; *n* = 4-6 in D).

**sFigure 4.** Uncropped gel images of Western blots seen in Figure 7A. Two images with different brightness adjustments (25% and 50%) are shown.

**sFigure 5.** Uncropped gel images of Western blots seen in Figure 7C. Two images with different brightness adjustments (25% and 50%) are shown.

**Supplementary Figure 1**

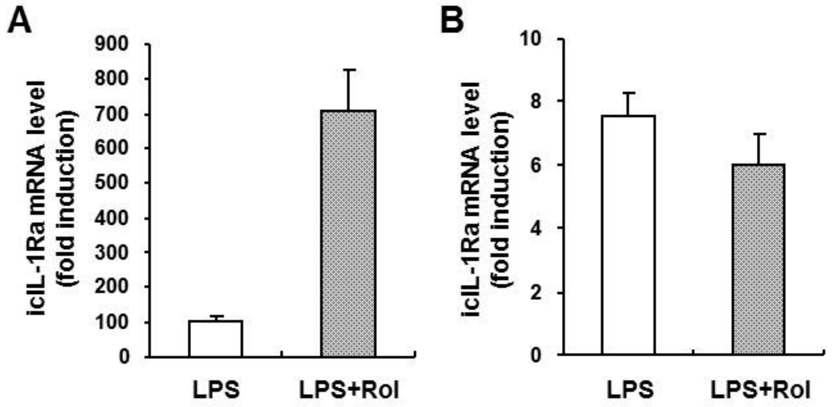


**Supplementary Figure 2**

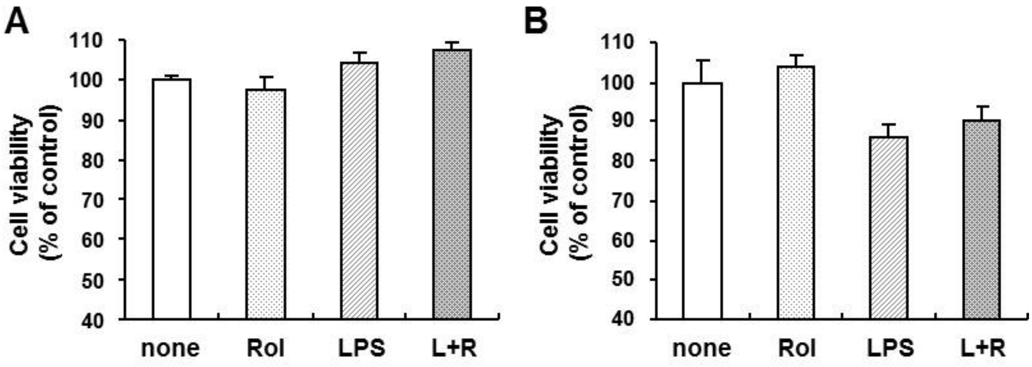

**Supplementary Figure 3**

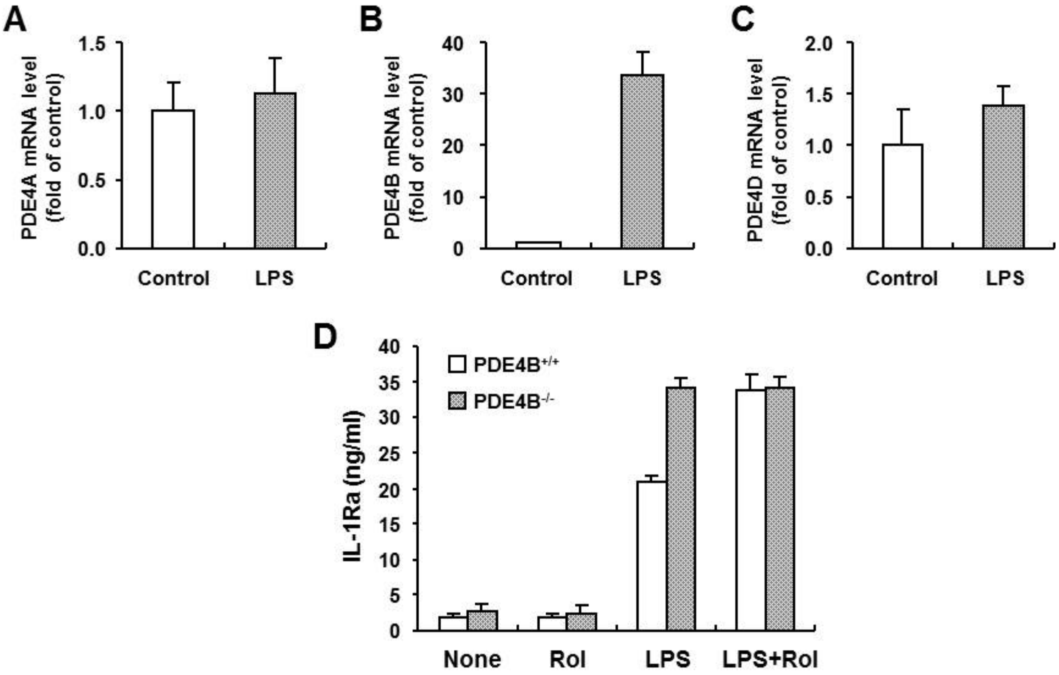

**Supplementary Figure 4**

25% brightness adjustment

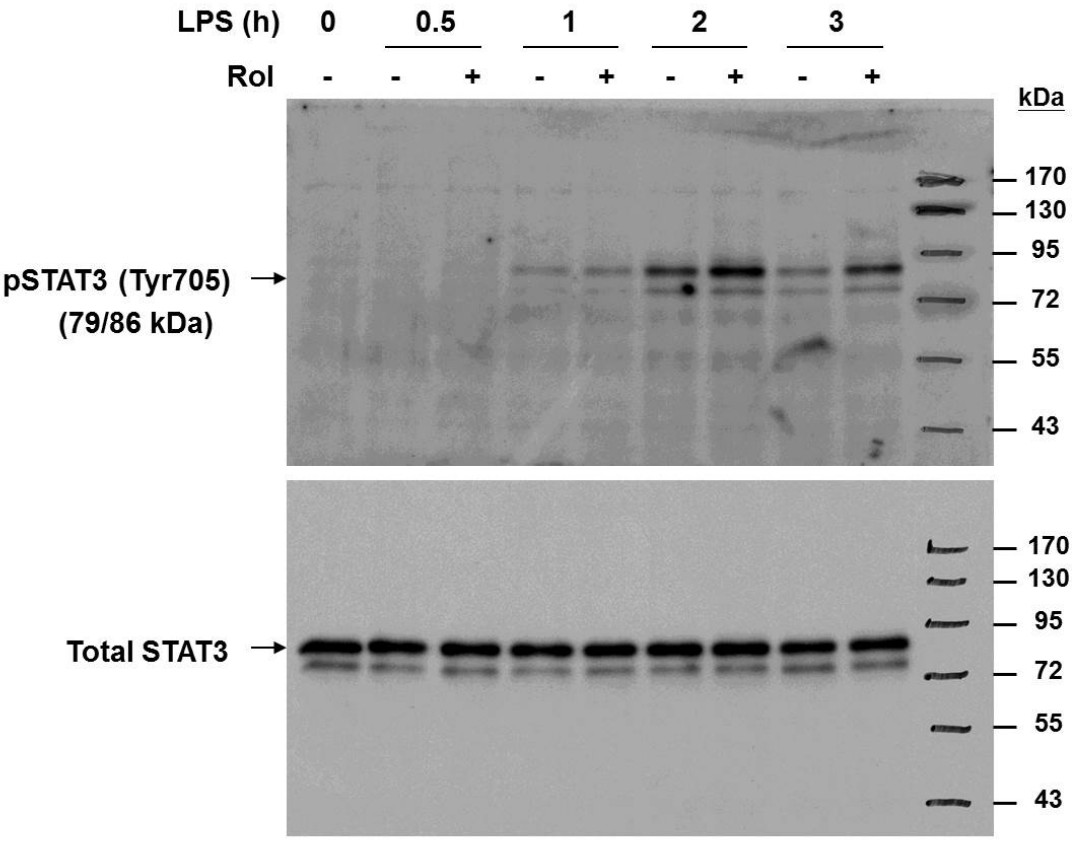


Adjust 25% brightness

**Supplementary Figure 4**

50% brightness adjustment

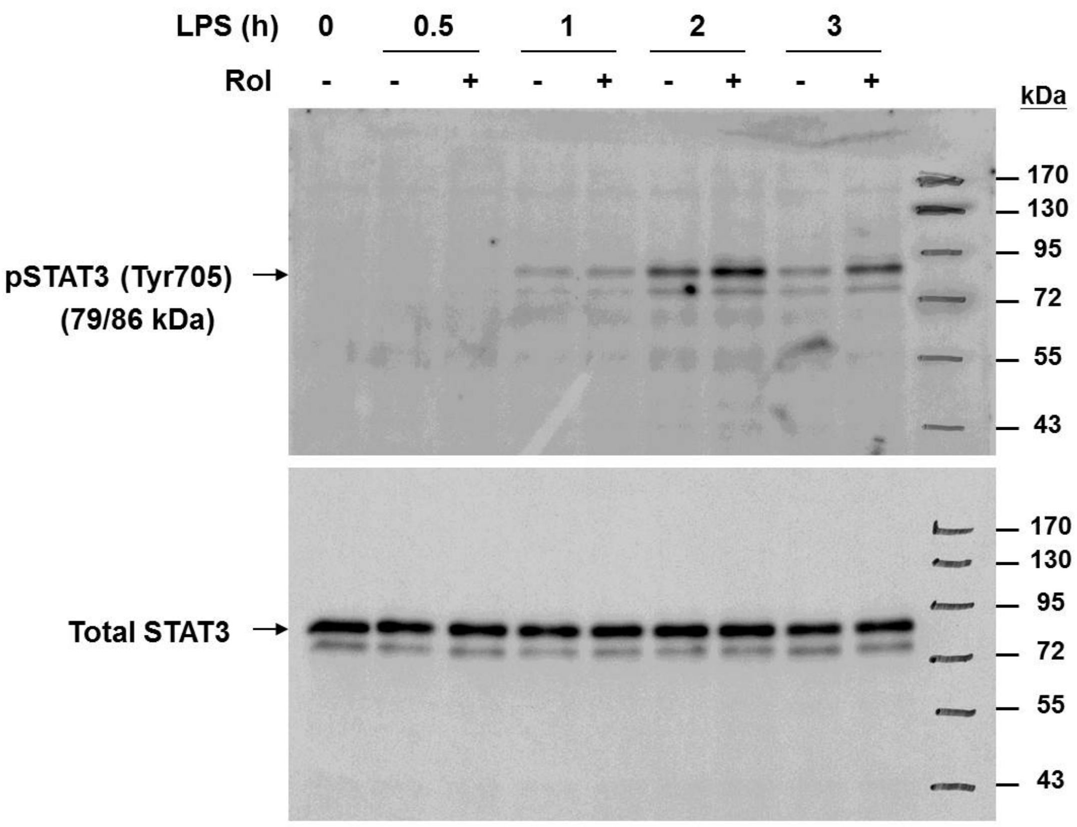


Adjust 50% brightness

**Supplementary Figure 5**

25% brightness adjustment

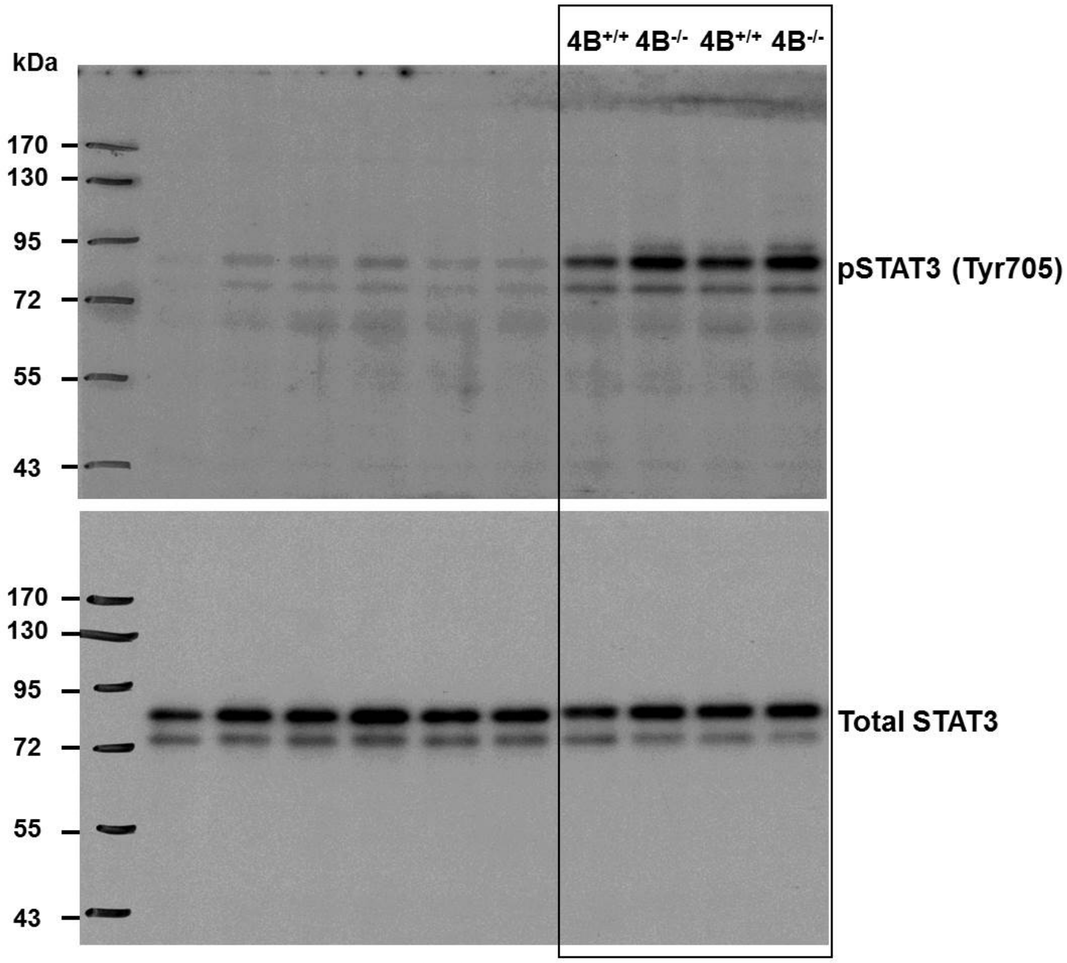


Adjust 25% brightness

**Supplementary Figure 5**

50% brightness adjustment

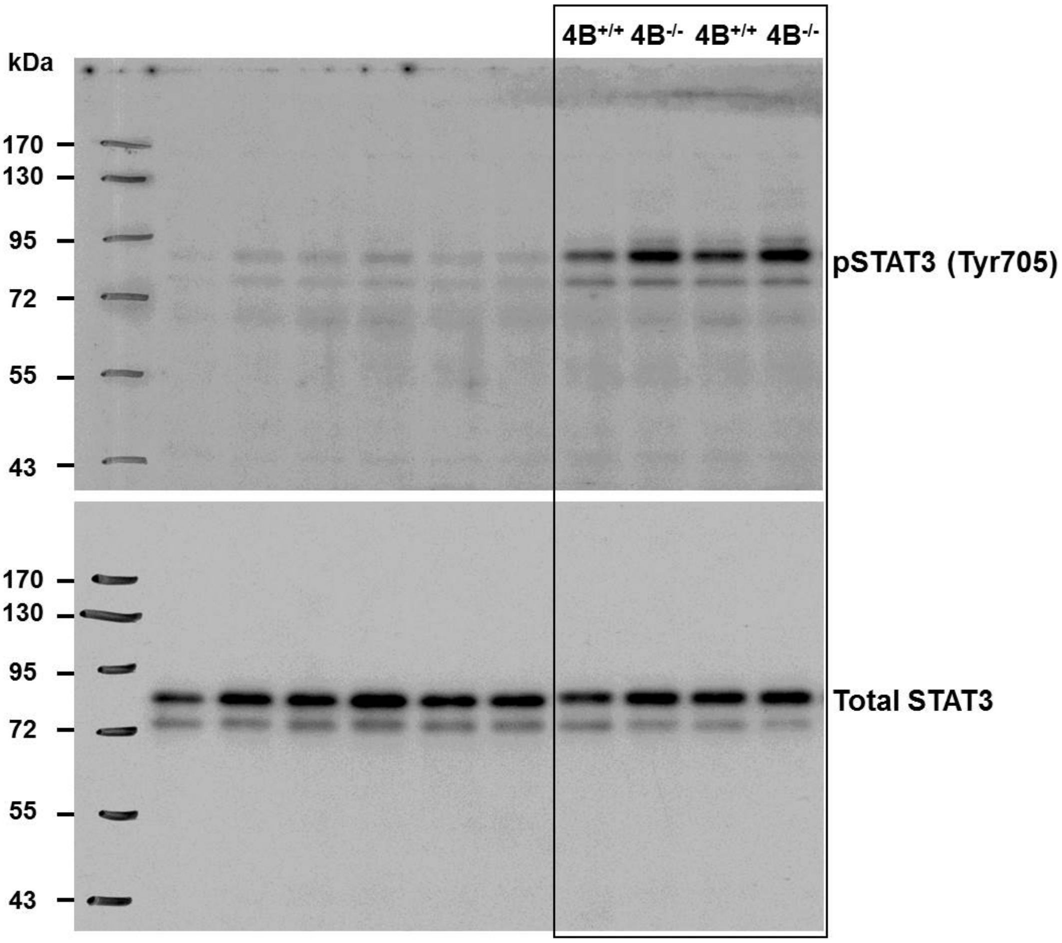


Adjust 50% brightness
